# Species dependent impact of helminth-derived antigens on human macrophages infected with *Mycobacterium tuberculosis*: Direct effect on the innate anti-mycobacterial response

**DOI:** 10.1371/journal.pntd.0005390

**Published:** 2017-02-13

**Authors:** Naomi Aira, Anna-Maria Andersson, Susmita K. Singh, Derek M. McKay, Robert Blomgran

**Affiliations:** 1 Division of Medical Microbiology, Department of Clinical and Experimental Medicine, Faculty of Health Sciences, Linköping University, Linköping, Sweden; 2 Gastrointestinal Research Group, Department of Physiology and Pharmacology, Snyder Institute for Chronic Disease, Cumming School of Medicine, University of Calgary, Calgary, Alberta, Canada; Universidade Federal de Minas Gerais, BRAZIL

## Abstract

**Background:**

In countries with a high prevalence of tuberculosis there is high coincident of helminth infections that might worsen disease outcome. While *Mycobacterium tuberculosis* (Mtb) gives rise to a pro-inflammatory Th1 response, a Th2 response is typical of helminth infections. A strong Th2 response has been associated with decreased protection against tuberculosis.

**Principal findings:**

We investigated the direct effect of helminth-derived antigens on human macrophages, hypothesizing that helminths would render macrophages less capable of controlling Mtb. Measuring cytokine output, macrophage surface markers with flow cytometry, and assessing bacterial replication and phagosomal maturation revealed that antigens from different species of helminth directly affect macrophage responses to Mtb. Antigens from the tapeworm *Hymenolepis diminuta* and the nematode *Trichuris muris* caused an anti-inflammatory response with M2-type polarization, reduced macrophage phagosome maturation and ability to activate T cells, along with increased Mtb burden, especially in *T*. *muris* exposed cells which also induced the highest IL-10 production upon co-infection. However, antigens from the trematode *Schistosoma mansoni* had the opposite effect causing a decrease in IL-10 production, M1-type polarization and increased control of Mtb.

**Conclusion:**

We conclude that, independent of any adaptive immune response, infection with helminth parasites, in a species-specific manner can influence the outcome of tuberculosis by either enhancing or diminishing the bactericidal function of macrophages.

## Author summary

The innate immune system is the first response against invading pathogens like the bacterium *Mycobacterium tuberculosis* (Mtb) or parasitic worms (helminths). The adaptive immune response takes over after being primed by the innate immune response. Infection with *Mycobacterium tuberculosis* typically gives rise to a pro-inflammatory T-helper(Th)-1 response while helminths promote a Th2 response which is needed to combat the infection. Co-infection with both of these pathogens could lead to reduced immunity contributing to worsening of tuberculosis due to an increased Th2 response caused by helminths. We found that antigens from different helminth species (a nematode, a cestode and a trematode) caused different responses towards Mtb in macrophages. Depending on the helminth species, the macrophages can be more or less capable of combating Mtb infection and priming the adaptive immune response, which in turn would influence the outcome of tuberculosis. Thus, exposure to helminth antigens, in a species-dependent manner, could lead to a better control of Mtb infection or worsening of tuberculosis.

## Introduction

Infection with helminth parasites and microbial pathogens present very different challenges to the mammalian immune system, and distinct immune effector mechanisms have evolved to combat infection with these different classes of organisms. Typically, infection with microbial pathogens requires the mobilization of professional phagocytes and Th1-dominated immunity, whilst some of these effectors may play a role in the response to helminth parasites, effective eradication of metazoans is the remit of Th2 immunity and its effectors, such as antibody, mucus and eosinophils [1–3]. The geographic distribution of tuberculosis (TB) and endemic helminth infections are almost superimposable and many individuals with TB will be, or will have been, infected with helminth parasites [3–5]. Given the general paradigm of the reciprocal inhibition of Th1 and Th2 immune responses and increase in TB globally, a comprehensive understanding of the impact of infection with helminth parasites on the response to *Mycobacterium tuberculosis* (Mtb) and the outcome of TB is essential.

Co-infection with helminth parasites and Mtb in mice and analysis of co-infected individuals has provided important, and often contrasting data, which may reflect host-parasite specificity in response to the helminths. For example, it was shown that mice infected with helminths and *M*. *tuberculosis* had a greater bacterial burden in their lungs [6–8], and contrarily, early control of *M*. *bovis* BCG in the lungs has been reported in helminth co-infected mice [9]. Where co-infection was shown to enhance susceptibility to TB, increased Th2 cytokines were implicated [6–8]; as for instance in the case of IL-4 promoting alternatively activated macrophages (AAMs) that accumulated in the lungs, correlating with deficient innate anti-tuberculosis protection [8]. Similarly, AAMs were found to be less effective than IFN-γ-treated macrophages in controlling *M*. *tuberculosis* [10]. Although no specific microbicidal mechanism was defined, it is likely that the polarization status of macrophages in helminth-infected mice affects the outcome of concomitant mycobacterial infection. Furthermore, helminth-derived products can directly reduce the LPS activation of macrophages, decreasing the expression or levels of pro-inflammatory cytokines [11–15]. Although the effect of helminth products in the context of macrophage Mtb infection was not tested those findings strengthen the notion that helminths, even without the amplification or signals via the adaptive immune response, could directly stimulate a regulatory M2-like macrophage that has suppressed mycobactericidal properties.

Many clinical studies indicate that infection with helminths modulate an individual’s susceptibility to TB, by, for example, increasing the risk of becoming latently infected with Mtb [16], and co-infected patients often present with more advanced disease [17]. Clinical studies of helminth-*M*. *tuberculosis* co-infection have focused mainly on documenting (often at a single time-point) the levels of Th1 and Th2 cytokines, and that helminth-induced Th2 polarization ultimately reduces cellular immunity to *M*. *tuberculosis* [17,18]. While murine models highlight macrophages as an important target cell affected during helminth-mycobacterial co-infection [9,19], the direct microbicidal mechanisms of human macrophages have not been studied. A number of scenarios would allow helminth-derived antigens (i.e. secretory/excretory (E/S) products) and mycobacteria to access the same macrophage: some intestinal helminths migrate through the lung as part of their life cycle; E/S products liberated from gastrointestinal helminths, filarial worms in lymphatics or *Schistosoma* species in the blood vessels would facilitate local and systemic delivery of antigens; and, the common mucosal immune system allows for the potential of phagocytes in the gut to traffic to the airways [20,21].

Hypothesizing that human monocyte-derived macrophages (hMDMs) co-exposed to *M*. *tuberculosis* and helminth antigens would have a decreased ability to handle the bacteria, a series of investigations were performed with antigens from a nematode (*Trichuris muris*, T.m), a cestode (*Hymenolepis diminuta*, H.d) and a trematode (i.e. *Schistosoma mansoni*, S.m)–representatives of the major groups of helminth parasites that infect humans. hMDMs co-treated with H.d or T.m displayed a decrease in the maturation of *M*. *tuberculosis*-phagosomes although, remarkably, after 1h of pre-exposure with the antigens, the number of intracellular bacteria were not different between hMDMs treated with *M*. *tuberculosis* ± worm antigen; however, after 48h of pre-exposure the co-treated hMDMs had increased bacterial burdens. Thus, extending the work of others showing that helminth antigens can directly affect macrophage function, we demonstrate that antigens from specific helminth parasites diminishes the bactericidal functions of human macrophages against *M*. *tuberculosis*, and that this effect occurs independent of any adaptive immune response.

## Materials and methods

### Ethics statement

Monocytes were obtained from heparinized peripheral human blood (Linköping University Hospital Blood Bank) from healthy donors who had given written consent for research use of the donated blood. Blood donation is classified as negligible risk to the donors and only anonymized samples were delivered to the researchers in accordance with the Declaration of Helsinki, not requiring a specific ethical approval according to paragraph 4 of Swedish law (2003:460) on Ethical Conduct in Human Research.

### Human Monocytes-Derived Macrophages (hMDMs)

Whole blood was added onto a density gradient and centrifuged for 40 min at 480xg at room temperature. The layer of peripheral blood mononuclear cells (PBMCs) was collected, washed and seeded into flasks to adhere for 1-2h before the lymphocytes were washed away. The adherent monocytes were left to differentiate into macrophages for 7 to 9 days in DMEM containing 10% pooled non-heat inactivated natural human serum, with full medium change twice during the culture period giving a mature macrophage population [22]. To confirm that the monocytes had differentiated into macrophages and not into dendritic cells, CD1c (dendritic cell marker) and CD14 staining was routinely performed (along with CD209, CD1a, and other markers). With this macrophage protocol cells were CD14^high^ and less than 2% expressing CD1c. Prior to experiments the hMDMs were seeded into either 96-well plates or 24-well plates containing cover slips.

### Establishment of Mtb Ag-specific CD4 T cell lines

For creating Mtb-specific CD4+ T cells from naïve CD4+ T cells, in order to analyze changes in antigen presentation from Mtb infected cells, we utilized our recently established system [23] using either the entire culture filtrate from H37Rv (e.g. PPD, purified protein derivative) or purified Ag85B protein, the major secretory and highly immunogenic protein of Mtb [24,25]. To generate antigen presenting cells (APCs) freshly isolated monocytes were cultured in RPMI-1640 containing 5% heat inactivated human AB serum supplemented with rhGM-CSF (10 ng/ml) and rhIL-4 (10 ng/ml) (both from Peprotech, USA) for 3 days. The APCs generated in this way were CD1a/c+. For T cell priming the generated APCs were harvested and γ-irradiated (25 Gy) before being co-incubated with naïve CD4 T-cells purified from PBMCs of the same donors using the human naive CD4+ T-cell isolation kit (Stem Cell Technologies), according to instructions provided by the manufacturer. Naïve CD4+ T cells (1x10^6^/well) were co-cultured with APCs (2.5x10^5^/well) in a 24-well plate and were stimulated with 10 μg/ml of purified protein derivative (PPD; culture filtrates from Mtb strain H37Rv obtained from the Staten Serum Institute, CPH, Denmark) or purified Ag85B protein (Rv1886c from Mtb strain H37Rv obtained from BEI Resources, Manassas, USA). Fresh media supplemented with IL-2 (20 IU/ml) were replenished once a week. The specificity test was carried out 3–4 weeks after generation of the CD4+ T-cell lines. For that, the CD4+ T cells and thawed autologous APCs were co-cultured at a 5:1 ratio along with PPD (10 μg/ml) or Ag85B (10 μg/ml) or Staphylococcal enterotoxin B (SEB; 1 μg/ml, used as a positive control) or ovalbumin (10 μg/ml, used as background control), and the level of IFN-γ in cell free supernatant was analyzed after 48h.

### Helminthic antigens

**Fig 1:**
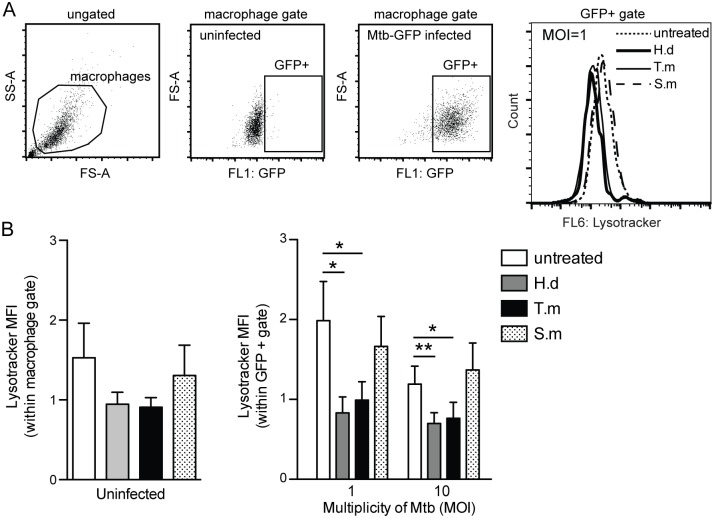
*H*. *diminuta* and *T*. *muris* antigens decrease acidification in Mtb infected macrophages. Human monocyte-derived macrophages (hMDMs) were treated for 1h ± antigens from *H*. *diminuta* (H.d; 100 μg/ml), *T*. *muris* (T.m; 50 μg/ml), or *S*. *mansoni* soluble egg antigen (S.m; 50 μg/ml), before being infected with green fluorescence protein (GFP)-expressing Mtb at different multiplicity of infection (MOI) for 2h. After infection, hMDMs were stained with LysoTracker Deep Red (LTDR). (A) Shows the gating strategy and representative LTDR-histograms for infected hMDMs ± antigens. (B) Results show the MFI-values of the LTDR signal in uninfected to the left, and infected macrophages (GFP-positive macrophages) to the right using flow cytometry. Data are presented as means *±* SEM from 6 independent hMDM donors. p*<0.05, p**<0.01 using One-way ANOVA.

**Fig 2:**
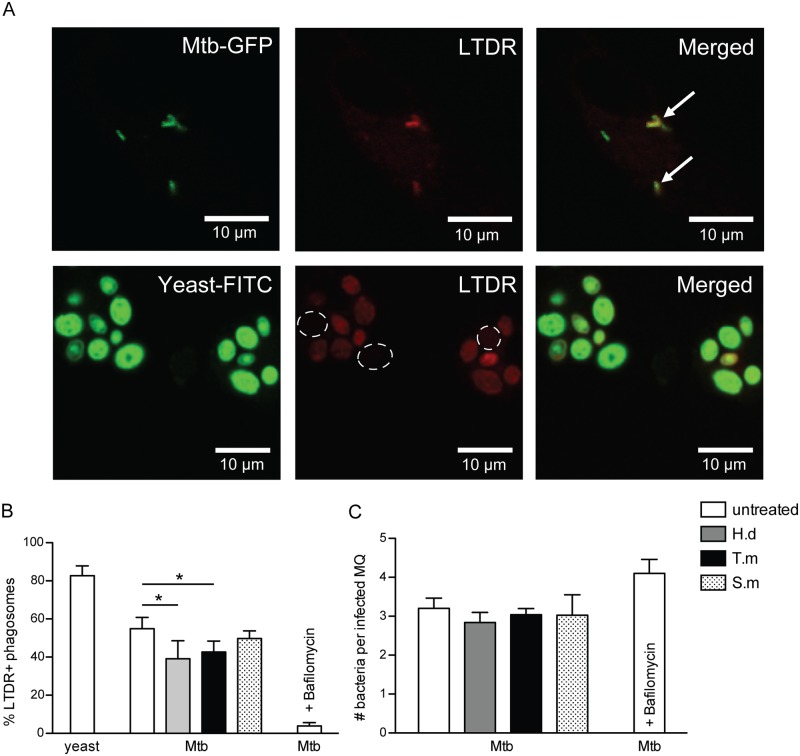
Co-localization of LysoTracker and Mtb-phagosomes is decreased by *H*. *diminuta* and *T*. *muris* antigen treatment. hMDMs were treated for 1h ± antigens from *H*. *diminuta* (H.d; 100 μg/ml), *T*. *muris* (T.m; 50 μg/ml), or *S*. *mansoni* soluble egg antigen (S.m; 50 μg/ml), before being infected with green fluorescence protein (GFP)-expressing Mtb (MOI = 1) for 4h. hMDMs were stained with LysoTracker Deep Red (LTDR). FITC-labeled yeast was used as a positive control. Bafilomycin (100 nM) was used as an inhibitor of acidification (negative control). (A) Representative micrographs displaying co-localization of LTDR and Mtb-GFP, shown by arrows. Since LTDR co-localized to most FITC-yeast phagosomes, absence of LTDR-staining is instead indicated by dashed circles. (B) Percentage LTDR^+^ phagosomes, counting 50–100 phagosomes/stimuli per donor, data expressed as means *±* SEM from n independent hMDM donors (n = 5 for yeast and n = 7 for Mtb ± bafilomycin). (C) Shows number (#) of bacteria per infected macrophage (MQ) (n = 7). p*<0.05 using One-way ANOVA.

**Fig 3:**
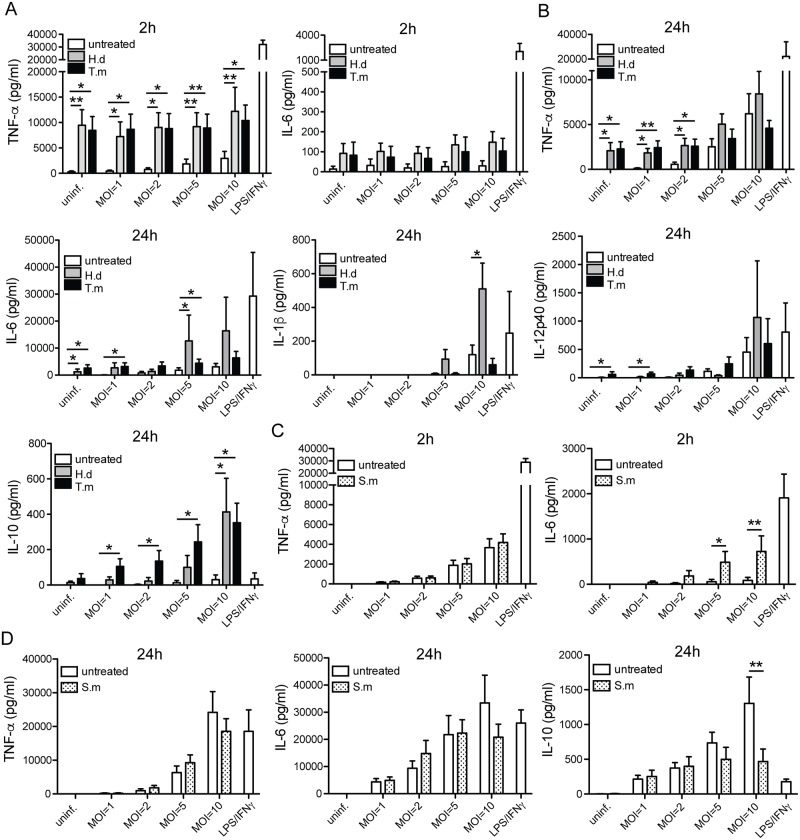
*H*. *diminuta* and *T*. *muris* antigen exposure stimulates early pro-inflammatory and late anti-inflammatory cytokine release. hMDMs were treated for 1h ± antigens from *H*. *diminuta* (H.d; 100 μg/ml), *T*. *muris* (T.m; 50 μg/ml), or *S*. *mansoni* soluble egg antigen (S.m; 50 μg/ml), before being infected with Mtb for 2h or 24h at the indicated MOIs. LPS/IFN-γ treatment was used as a positive pro-inflammatory stimulus. (A) TNF-α and IL-6 secretion at 2h with/without H.d or T.m. (B) TNF-α, IL-6, IL-1β, IL-12p40 and IL-10 at 24h with/without H.d or T.m. Data are presented as means *±* SEM. n = 7 independent hMDM donors for 2h measurements and n = 6 for 24h measurements, and n = 5–7 for IL-10. p*<0.05, p**<0.01 using One-way ANOVA. S.m data in (C; 2h) and (D; 24h) are presented as means *±* SEM from 7 independent donors that are different from the donors presented in (A) and (B), therefore separate graphs. p*≤0.05, p**≤0.01 using paired Student t-test.

**Fig 4:**
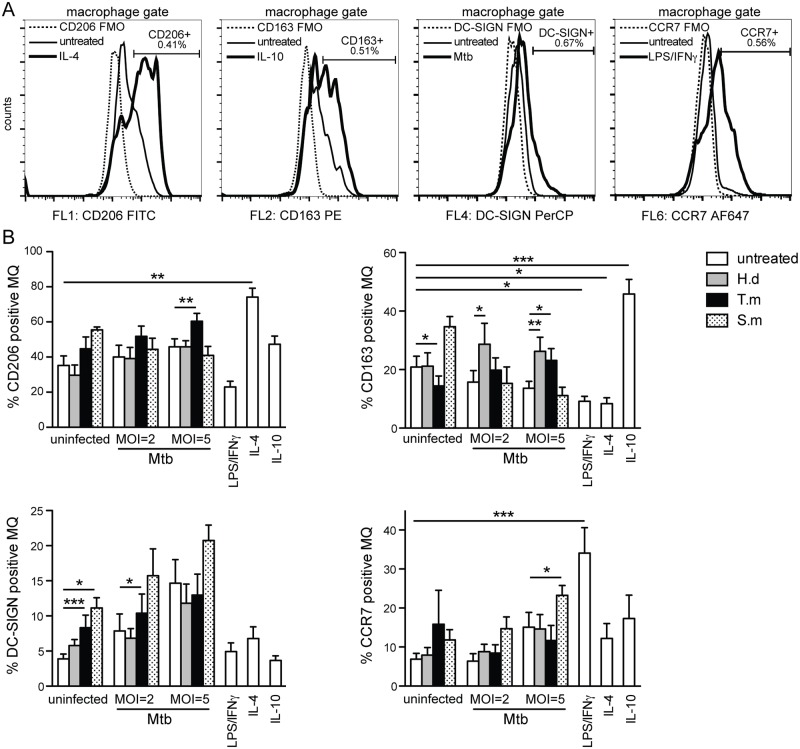
Helminth antigen species-specific polarization of hMDMs upon co-exposure with Mtb. hMDMs were treated for 1h ± antigens from *H*. *diminuta* (H.d; 100 μg/ml), *T*. *muris* (T.m; 50 μg/ml), or *S*. *mansoni* soluble egg antigen (S.m; 50 μg/ml), before being infected with Mtb at the indicated MOI. After 24h hMDMs were detached and stained with a combination of CD206-FITC (M2a marker), CD163-PE (M2c marker), CCR7-AF647 (M1 marker), or DC-SIGN-PerCP (regulatory macrophage phenotype). LPS/IFN-γ was used as a positive control for M1-stimuli, IL-4 as M2a-stimuli, and IL-10 as M2c-stimuli. (A) Representative histograms showing the individual fluorescence minus one (FMO) controls (with indicated %) that each positive gate was based on, and a showing signal profile from untreated together with either a positive stimulus or Mtb infection. (B) Data are expressed as means *±* SEM from 8 independent hMDM donors. p*<0.05, p**<0.01, p***<0.001 using One-way ANOVA.

**Fig 5:**
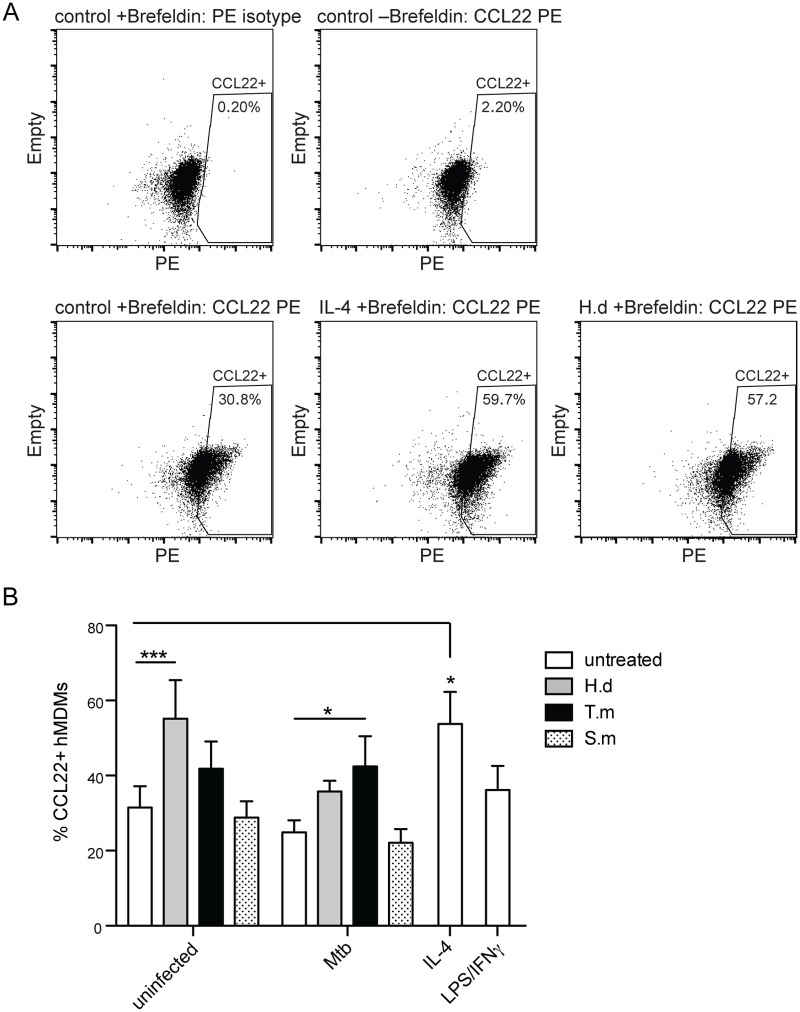
*H*. *diminuta* antigen and *T*. *muris* antigen co-exposure with Mtb stimulates increased CCL22 protein production in hMDMs. hMDMs were treated for 1h ± antigens from *H*. *diminuta* (H.d; 100 μg/ml), *T*. *muris* (T.m; 50 μg/ml), or *S*. *mansoni* soluble egg antigen (S.m; 50 μg/ml), before being infected with Mtb at MOI = 5 for 24h. Brefeldin A was added 2h after Mtb infection to retain intracellularly produced proteins. After 24h of infection cytofix/cytoperm treated cells were stained intracellularly for flow cytometry using anti-CCL22 PE or a PE isotype control antibody to set the CCL22+ gate for data shown in (B). (A) Representative dot plots of the entire macrophage population (macrophage gate depicted in Fig 1A) showing the PE-profile of control cells treated with brefeldin A and stained with PE isotype control antibody (upper left) that is used to set the gate for % CCL22+ hMDMs, also illustrating PE-profile for CCL22 stained control (untreated), IL-4 and H.d treated hMDMs. (B) Data are expressed as means *±* SEM from 5 independent hMDM donors. p*<0.05, p***<0.001 using One-way ANOVA.

**Fig 6:**
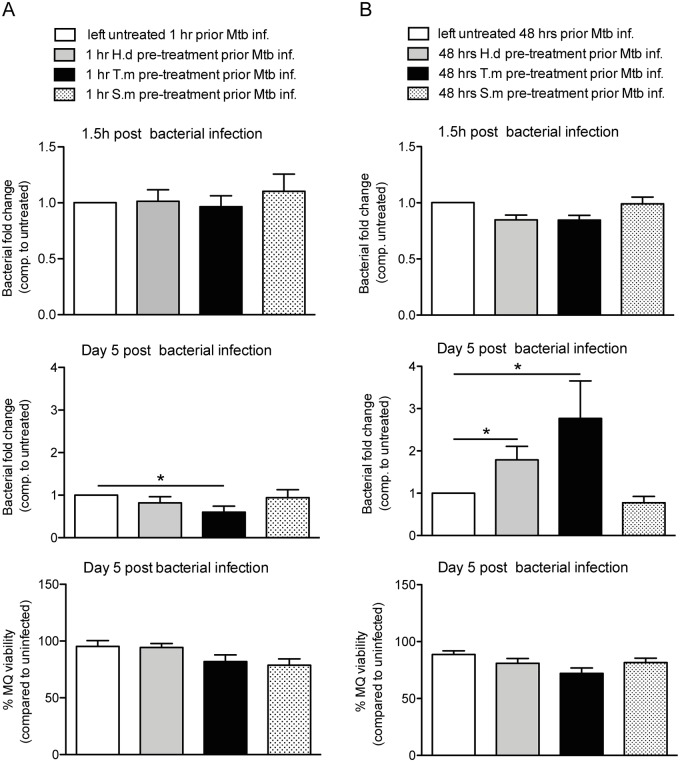
Mimicking chronic helminth infection by long-term antigen exposure results in lost hMDM control of Mtb. hMDMs were treated with helminth antigens from *H*. *diminuta* (H.d; 1.5 μg/ml), *T*. *muris* (T.m; 1.5 μg/ml), or *S*. *mansoni* soluble egg antigen (S.m; 1.5 μg/ml) for 1h (A) or 48h (B) prior to infection for 1.5h with luciferase expressing Mtb (MOI = 2). Extracellular bacteria were washed away and Mtb phagocytosis was evaluated (upper horizontal panels), or washed hMDMs were incubated for 5 days at 37°C before the bacterial fold change was determined (middle horizontal panels). hMDMs pretreated with antigens for 1h prior Mtb infection had continuous presence of antigens throughout the experiment (A), whereas hMDMs pretreated for 48h received antigens prior Mtb infection only (B). A and B show the total bacteria (combined luciferase signal from hMDM lysate and supernatant) compared to untreated (only Mtb), data expressed as means *±* SEM from 4 independent hMDM donors. Bottom horizontal panel, show hMDM viability after 5 days post Mtb infection measured using calcein AM uptake before lysates were generated for middle horizontal panels. Viability data are normalized against uninfected hMDMs set to 100%. p*<0.05 using One-way ANOVA.

**Fig 7:**
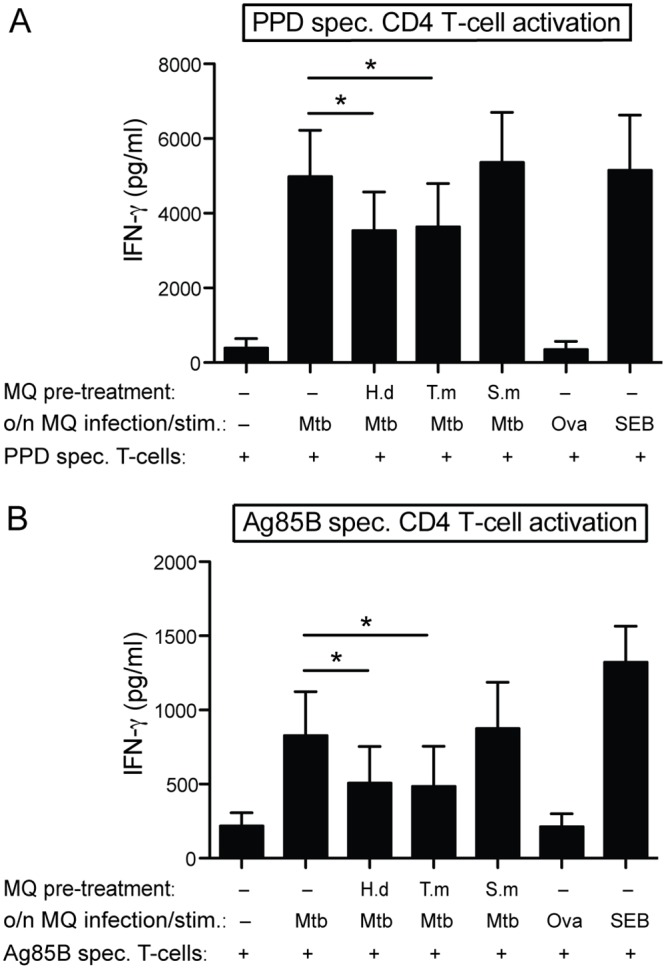
*H*. *diminuta* and *T*. *muris* antigen treated hMDMs reduce Mtb-specific antigen presentation to CD4+ T cells. hMDMs were left untreated, or treated for 48h with 10 μg/ml of *H*. *diminuta* (H.d), *T*. *muris* (T.m), or *S*. *mansoni* soluble egg antigen (S.m). Thereafter hMDMs were either infected with Mtb (MOI = 5) or stimulated with PPD or SEB (positive controls) or ovalbumin (Ova; background control) for 24h, before being co-cultured with autologous PPD-specific (A) or Ag85B-specific (B) CD4^+^ T cells (1:5 DC:T cell ratio). Cell free culture supernatants were collected 48h later, and assayed for IFN-γ, data expressed as means *±* SEM from 7 independent hMDM donors. p*<0.05 using One-way ANOVA.

**Fig 8:**
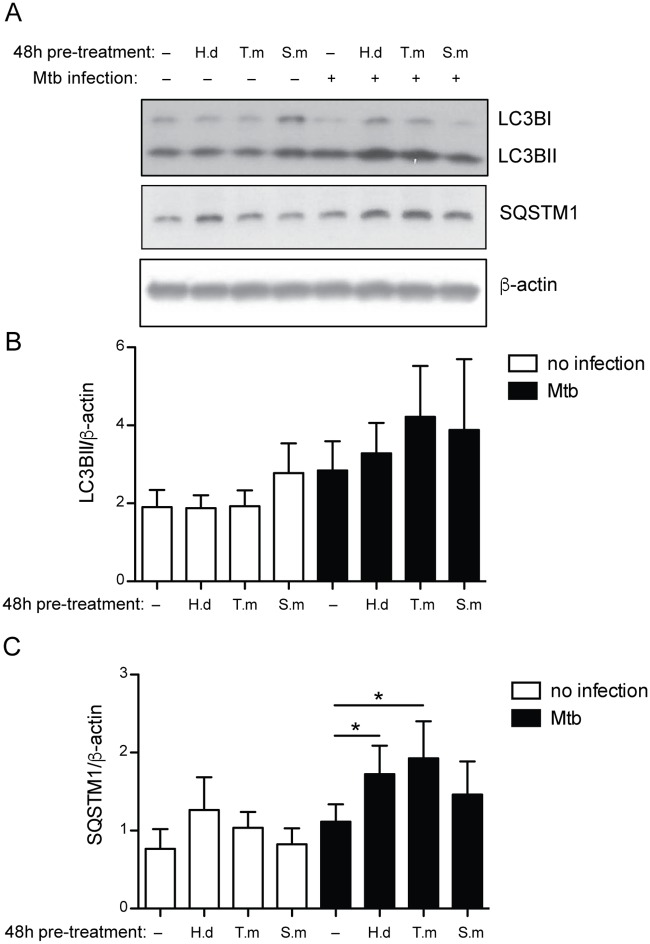
*H*. *diminuta* and *T*. *muris* antigen treated hMDMs infected with Mtb show accumulation of autophagy proteins. hMDMs were left untreated, or treated for 48h with 10 μg/ml of *H*. *diminuta* (H.d), *T*. *muris* (T.m), or *S*. *mansoni* soluble egg antigen (S.m), before being infected with Mtb (MOI = 2). After 4.5h of infection the cellular lysates were subjected to western blot using anti-LC3B, or anti-SQSTM1 antibodies, and anti-β-actin antibody as a loading control. Representative western blot images of LC3B, SQSTM1 and β-actin (A). Densitometry analysis of LC3BII (B) and SQSTM1(C), normalized against β-actin. Data are expressed as mean ± SEM of 4 independent hMDM donors. p*<0.05 using One-way ANOVA.

The *H*. *diminuta* antigens were prepared from whole worm crude extract [26], and *T*. *muris* antigens (a kind gift from Dr. W. Khan (McMaster Uni. Hamilton, ON, Canada)) from excretory and secretory products (E/S) from the worm into 5% PenStrep containing medium [27]. *S*. *mansoni* soluble egg antigen was from Professor Mike Doenhoff, Nottingham University, Nottingham UK. Stock concentrations of antigens in PBS were confirmed by Bradford assay and aliquots stored at -80°C until use. The hMDMs were treated with the helminth antigens at the concentrations indicated in the legends, starting with concentrations previously used for *H*. *diminuta* (100 μg/ml; [26]) and *T*. *muris* (50 μg/ml; [11]). Antigens were added 1h (Figs 1–6) or 48h (Figs 6–8) prior to infection with *M*. *tuberculosis*, as specifically stated in the figure legends. Since concentrations above 3 μg/ml of *T*. *muris* had a direct mycobactericidal effect, 1.5 μg/ml across all antigens were chosen when evaluating the intra-macrophage killing capacity of Mtb. All helminth antigens were free from LPS contamination, i.e. found below the detection limit of Pierce LAL Chromogenic Endotoxin Quantification Kit using 1 mg/ml of the individual antigens.

### Bacterial preparation and infection of hMDMs

*M*. *tuberculosis* (Mtb) H37Ra or H37Rv (only for Fig 7) were grown at 37°C in Middlebrook 7H9 broth supplemented with 0.05% Tween-80 and 10% ADC enrichment (Becton Dickinson) with 20 μg/ml kanamycin for green fluorescence protein (GFP)-expressing Mtb or 100 μg/ml hygromycin for luciferase-expressing Mtb. Log phase bacterial cultures was centrifuged two times for 5 min at 5000xg and the bacteria were separated by needle shearing first in PBS-Tween-80 (0.05%) and then in serum-free medium. The OD value was measured to determine the concentration and for calculation of multiplicity of infection (MOI). After pretreatment with the antigens, Mtb was added to the hMDMs at different MOI (1, 2, 5 or 10) and time-points as indicated in the figure legends. Antigen presentation experiments (Fig 7) were performed with H37Rv that was heat inactivated for 1h at 70°C prior use; for all other experiments, live H37Ra was used. We have recently shown that both *M*. *tuberculosis* H37Ra and H37Rv infect and can replicate in hMDMs (although H37Rv replicate to a greater extent), and that both the strains can manipulate/block the autophagy pathway [28].

### LysoTracker staining

After 1h pretreatment with antigens and 2h infection with GFP-Mtb, LysoTracker deep red (LTDR) (Life technologies) was added to a final concentration of 75 nM for 30 min to visualize acidic compartments. The cells were detached with accutase (Stem Pro accutase, Gibco by life technologies) and fixed in 4% PFA before being run in a Gallios flow cytometer (Beckman coulter). The quantitative LTDR signals were analyzed with Kaluza software version 1.2 (Beckman Coulter, USA).

Qualitative assessment of phagosomal acidification using LTDR was performed with hMDMs adhered to coverslips [28]. hMDMs were pretreated with helminth antigens for 1h before infection with GFP-Mtb, or FITC-labelled yeast as a positive control for phagolysosomal maturation. Negative controls were pretreated with 100 nM bafilomycin (from *streptomyces griseus*, Sigma Aldrich) prior to infection with Mtb. LTDR was added for the last 2h of the 4h Mtb infection before fixation and staining with wheat-germ agglutinin (WGA) AF350 (Life technologies) 1 μg/ml for 20 sec followed by mounting. All cover slips were analyzed in a LSM 700 Zeiss inverted confocal microscope and the images were observed in a blinded manner. The brightness of the images was increased for visualization purposes only, after the completion of the analysis.

### Cytokine analysis

Helminth treated hMDMs were infected with Mtb at different MOI for 2h or 24h. Time-points for analyzing cytokine secretion was based on our previous analysis for pro- and anti-inflammatory cytokines secreted from stimulated hMDMs (including Mtb exposure), were TNF-α was secreted as early as 2h after exposure whereas other cytokines were more robustly secreted and detected after 18-24h of stimulation [29]. The medium supernatants from triplicate wells of each treatment were pooled, cleared from cellular debris, and stored in -70°C until assayed. TNF-α, IL-12p40, IL-6, IL-1β, and IL-10 levels were determined by cytometric bead array analysis, performed according to the manufacturer’s protocol (BD Biosciences). Detection of cytokines was performed by flow cytometry (Becton Dickinson) and cytokine concentrations were analyzed using Kaluza software version 1.2 (Beckman Coulter, USA).

### Staining for M1/M2 macrophages

hMDMs exposed to helminth antigens and infected with different MOI of Mtb were stained with different macrophage polarization markers. For positive controls of M2 macrophages, IL-4 (20 ng/ml) or IL-10 (2 ng/ml) was added to induce M2a or M2c macrophages, respectively [30]. A cocktail of 100 ng/ml LPS and 100 U/ml IFN-γ was added as a positive control for induction of pro-inflammatory M1 macrophages. Stimulated and infected hMDMs were detached with accutase (Stem Pro accutase, Gibco by life technologies) and centrifuged at 900xg for 5 min prior to adding 10 μl DC-SIGN/CD209 PerCP for 15 min at room temperature (RT). Twenty μl of a mix of the antibodies; CD206-FITC, CD163-PE, CCR7-AF647, was added with the dilutions 1:26.6 for CCR7 and 1:6.6 for the rest and incubated for 25 min RT. Fluorescence minus one (FMO)-controls received an antibody mix lacking one of the respective antibodies. The samples were acquired on a Gallios flow cytometer (Beckman Coulter), and the hMDMs were analyzed using Kaluza software 1.2 where the % marker positive hMDMs were evaluated based on their respective FMO-control.

### Staining for CCL22+ macrophages

hMDMs exposed to helminth antigens were infected with Mtb (MOI = 5) for 24h and treated with brefeldin A (5μg/ml) the last 22h before being detached with trypsin EDTA and stained intracellularly using a PE mouse anti-human CCL22 antibody (clone T51-719) using Cytofix/Cytoperm according to the manufacturer’s protocol (BD Biosciences). A PE isotype matched control antibody was used at same concentration as the specific CCL22 antibody (4μg/ml; determined by titration) to set the positive gate. IL-4 and a LPS/IFN-γ cocktail was used as positive stimuli for M2 and M1 macrophage, respectively, at concentrations indicated above. The samples were acquired on a Gallios flow cytometer (Beckman Coulter), and the hMDMs were analyzed using the software FlowJo (version 10.1) where the % CCL22 positive hMDMs were evaluated based on gates set by the PE isotype control antibody.

### Cell viability and Mtb replication assay

After antigen treatment for 1h or 48h, Mtb expressing luciferase were added for 1.5h. Extracellular bacteria were washed away and new medium with re-added antigens was added and hMDMs incubated for 5 days. hMDM viability was measured by calcein-AM uptake, using 0.4% calcein in PBS incubated for 30 min, that was added after removal of supernatants and washing of cells. The calcein fluorescence was measured in a Modulus microplate reader prior to lysing of hMDMs to measure the bacteria in the lysate as described previously [31]. The luminescence from live bacteria in both supernatant and lysate was measured.

### Antigen presentation assay

hMDMs (3x10^4^/ well) were treated with helminth antigens (10 μg/ml of *H*.*d*, *T*.*m* or *S*.*m*) for 48h, thereafter stimulated with heat killed Mtb (MOI = 5), PPD, SEB, or ovalbumin for 24h. Supernatants were discarded and hMDMs were co-cultured with autologous Mtb antigen-specific (PPD or Ag85B) CD4+ T-cells (1.2x10^5^ cells/ well). Cell free supernatants were examined for IFN-γ production after 48h of co-culture.

### Western blot

hMDMs were untreated or treated with 10 μg/ml of the antigens for 48h prior to Mtb infection for 4.5h. The cells were lysed in boiling 2x Laemmli sample buffer and western blot was run as previously described [28] using the primary antibodies rabbit monoclonal anti-LC3 (D11) (Cell signaling) diluted 1:5000, mouse monoclonal anti-SQSTM1 D-3 (Santa Cruz Biotechnology) diluted 1:1000 and mouse monoclonal anti-β-actin (clone AC-74, Sigma Aldrich) diluted 1:10 000. The secondary antibodies were polyclonal goat anti-rabbit and anti-mouse immunoglobulins/HRP (Dako Cytomation) diluted 1:2000 except for the one against anti-β-actin which was diluted 1:10 000. Inhibition in autophagosome maturation (i.e. functional autophagy) was evaluated based on increases in either the LC3BII/actin ratio or the SQSTM1/actin ratio according to our previous experience with *M*. *tuberculosis*-infected hMDMs [28].

### Statistics

Statistical analyses were performed with Graph pad prism (version 5.0f). Data from multiple treatments were analyzed using Repeated Measures ANOVA with Dunnett post test, and for single treatment a paired Student t-test was used as indicated. p values < 0.05 were considered significant.

## Results

### Helminth antigens decrease maturation of Mtb-phagosomes in hMDMs

To study the direct effect of helminth antigens on hMDMs function we first evaluated Mtb phagosomal maturation in macrophages simultaneously exposed to helminth antigens. The first approach was to quantitatively measure the lysosomal marker LysoTracker Deep Red (LTDR)-signal within uninfected and infected macrophages (the GFP^+^ fraction) using flow cytometry. The helminth antigens did not significantly affect the basal level of the LTDR-signal in uninfected hMDMs. In hMDMs treated with Mtb only, there was a clear decrease in LTDR-signal with increasing MOI of Mtb (Fig 1; and previously shown [32]), indicating that Mtb-infection alone blocks phagosome acidification. hMDMs infected with Mtb at MOI = 1 and co-exposed with antigens from *H*. *diminuta* or *T*. *muris* showed a ≥ 50% drop in LTDR MFI, compared to Mtb only infection (p < 0.05 for both *H*. *diminuta* and *T*. *muris*, respectively). Similar results were obtained at MOI = 10 with a 42% reduction in LTDR MFI for *H*. *diminuta* co-treated and 36% reduction for *T*. *muris* co-treated hMDMs (p < 0.05 for both *H*. *diminuta* and *T*. *muris* treatment). Thus, antigens from these helminths effectively suppress acidification and phagosomal maturation in Mtb infected hMDMs, irrespective of the bacterial burden. Schistosoma soluble egg antigen co-treatment did not affect the LTDR-signal at any MOI tested.

Using confocal microscopy the co-localization of the phagosomal maturation marker and the bacteria was analyzed (Fig 2A). Areas with phagosomes contributed to the strongest LTDR-signal, whereas remaining parts of the cells did not, indicating that the LTDR-signal measured by flow cytometry (Fig 1) is maturing phagosomes and not general hMDMs acidification. Bafilomycin, the v-ATPase inhibitor used as a negative control, strongly inhibited LTDR co-localization to Mtb-phagosomes (Fig 2B), further verifying the specificity of this probe. hMDMs ingesting FITC-labeled yeast, used as positive control for phagosome maturation (Fig 2B), showed a 1.5-fold increase in LTDR co-localization compared to that of Mtb phagosomes (from 54% with Mtb to 83% LTDR-positive phagosomes with yeast), consistent with Mtb virulence mechanisms being active in preventing phagosomal maturation (Fig 2B) [33]. Significantly less LTDR-Mtb co-localization was observed in macrophages co-exposed with *H*. *diminuta* (p < 0.05) or *T*. *muris* (p < 0.05) antigens. Thus, while Mtb can obstruct phagosomal maturation, concomitant exposure to helminth antigens can further reduce the capacity of hMDMs to handle and efficiently process Mtb phagosomes. Again, schistosoma soluble egg antigen co-treatment did not affect the Mtb-LTDR co-localization. No differences in number of intracellular Mtb were seen in helminth antigen treated or untreated hMDMs (Fig 2C), indicating that the reduced acidification and phagosome maturation was not due to differences in total bacterial uptake by the macrophages.

### *H*. *diminuta* and *T*. *muris* induce an early pro-inflammatory cytokine release followed by a late anti-inflammatory response with increased IL-10

Cytokine secretion was monitored in uninfected and infected hMDMs at increasing bacterial loads (MOI = 1, 2, 5, and 10) (Fig 3A–3D). We evaluated the early cytokine secretion at 2h, and the delayed cytokine secretion at 24h post-treatment/infection. Untreated uninfected hMDMs showed low secretion of TNF-α at 2h (<300 pg/ml), whereas *H*. *diminuta* and *T*. *muris* treatment of infected and uninfected hMDMs induced an immense TNF-α secretion (~9500 pg/ml and ~8500 pg/ml, p < 0.01 and p < 0.05 compared to untreated uninfected, respectively). After 24h, the levels of TNF-α had decreased in the *H*. *diminuta* and *T*. *muris*-treated cells although still exhibiting significant increase in uninfected and infected up to MOI = 2, but not for the higher MOIs were the Mtb-infected only cells had caught up with those of the co-exposed groups. The initial low levels of IL-6 at 2h (untreated <30 pg/ml, *H*. *diminuta* and *T*. *muris*-treated ≤150 pg/ml, irrespective of infection) had increased substantially at 24h showing a significant increase with helminth-treatment in uninfected hMDMs (p < 0.05 for both *H*. *diminuta* and *T*. *muris* treatment), and for *H*. *diminuta* or *T*. *muris* co-exposed hMDMs at MOI = 1(p < 0.05 for *T*. *muris* co-exposed) and MOI = 5 (p < 0.05 for both *H*. *diminuta* and *T*. *muris* co-exposed). Except for IL-6 and TNF-α no other cytokines measured showed significant release above background at 2h. Unlike the other cytokines measured, IL-1β was not secreted in any conditions under MOI = 5, and *H*. *diminuta* exhibited a strong augmenting effect on the Mtb-triggered response that was 14x-fold at MOI = 5 and 4.3x-fold at MOI = 10 (p < 0.05). Evaluating secretion of the anti-inflammatory cytokine IL-10, the helminthic antigens *H*. *diminuta* and *T*. *muris* exhibited a synergistic effect with increasing MOI of Mtb. From these analyses we conclude that *H*. *diminuta* and *T*. *muris* antigens can trigger an early pro-inflammatory response with increased TNF-α both in the absence and presence of Mtb-infection which is then shifted towards an anti-inflammatory response with a synergistic increase of IL-10. *S*. *mansoni*-antigen treatment of hMDMs did not induce any cytokine secretion by itself and did not augment the Mtb-induced TNF-α cytokine secretion (Fig 3C and 3D), but instead lowered the Mtb-induced IL-10 secretion (Fig 3D).

### Helminth antigen stimulation and Mtb co-infection induce distinct polarization of hMDMs

To elucidate whether helminth antigens, in the absence of polarizing modulators from the adaptive immune response, have the capacity to polarize human macrophages toward M1 (classically activated or pro-inflammatory) or M2 (alternatively activated or anti-inflammatory), hMDMs surface expression of CD206 (mannose receptor; M2a, indicative of IL-4 macrophages), CD163 (M2c, also referred to as IL-10 macrophages), CCR7 (M1-marker for macrophages), and DC-SIGN (marker for regulatory alternatively activated M2-macrophages) [30,34] were investigated (Fig 4). We used the same experimental setup and rational with increasing bacterial MOI as for detecting cytokine secretion. Of the several polarization patterns that the helminth antigens induced without or with infection, we focus on the dominant effect for each individual antigen during co-stimulation with Mtb. *T*. *muris* induced a polarization towards M2a-like (CD206^+^) hMDMs with elevated DC-SIGN expression. *H*. *diminuta* induced polarization towards M2c-like (CD163^+^) hMDMs. *S*. *mansoni* soluble egg antigen triggered increased DC-SIGN expression in combination with enhanced expression of the M1-marker CCR7 at the highest MOI. In the absence of antigen, CD206, DC-SIGN, and CCR7 expression was gradually elevated with increasing bacterial MOI, whereas CD163 decreased with increasing bacterial MOI. Despite Mtb being immunomodulatory on macrophage polarization itself, helminth-antigen exposure further exacerbated the Mtb effect as seen by the increasing expression of CD206 (*T*. *muris*), CD163 (*H*. *diminuta*), DC-SIGN (*T*. *muris* and *S*. *mansoni* soluble egg antigen), and CCR7 (*S*. *mansoni* soluble egg antigen).

### CCL22 chemokine expression indicating specific M2-polarization in *H*. *diminuta* and *T*. *muris* treated hMDMs

Expression of CCL22, being specifically expressed under M2-stimulation (e.g. IL-4) at 24h post-stimulation [35,36] was used to further decipher the shift in macrophage polarization upon helminth antigen challenge (Fig 5). The data for CCL22 indicate that antigen from *H*. *diminuta* and *T*. *muris* (but not from *S*. *mansoni*) significantly increase its production in uninfected (*H*. *diminuta*, p < 0.001) or in combination with *M*. *tuberculosis* infection (*T*. *muris*, p <0.05). Taken together with the surface expression of the M2a-marker (CD206; augmented by *T*. *muris* co-exposure) and the M2c-marker (CD163; augmented by both *T*. *muris* and *H*. *diminuta* co-exposure) this indicated that these antigens are driving polarization towards an AAM phenotype.

### Mimicking chronic helminth infection by long-term exposure prior to Mtb infection results in lost intracellular control of Mtb depending on helminth species

Our data thus far indicate that some helminth antigens can have both an early pro-inflammatory effect and a long-lasting immunoregulatory effect pertaining to polarization of cells towards an AAM phenotype with reduced phagolysosome fusion. To investigate these rather opposing effects on hMDMs ability to handle Mtb infection, hMDMs were exposed to helminth antigens for various periods before infected by Mtb. hMDMs were preincubated with helminth antigens for 1h to investigate the early effects of worm exposure, and for 48h to investigate the long-term or chronic effects of worm infection on Mtb infection. Neither exposure time affected the uptake of Mtb into hMDMs, nor the viability of hMDMs (Fig 6, upper and lower horizontal panel, respectively). Exposure with the *T*. *muris* antigen for 1h prior to infection with Mtb induced a slight, yet statistically significant (p < 0.05), decrease in the bacterial burden in hMDMs at 5 days post-infection (compared to untreated) (Fig 6A, middle panel). hMDMs exposure with helminth antigens for 48h prior to infection, instead caused a 2.8-fold increase (p < 0.05) in the bacterial burden for *T*. *muris* and 1.7-fold increase in the bacterial burden for *H*. *diminuta* (p < 0.05), whereas *S*. *mansoni* antigen induced increased control of Mtb showing a bacterial burden of 0.7-fold compared to untreated infected hMDMs (Fig 6B, middle panel). This suggests that during chronic helminth infection the direct immunomodulatory properties of helminthic antigens, can either facilitate growth of Mtb inside human macrophages, or help macrophages maintain control over Mtb depending on the helminth species.

### *H*. *diminuta* and *T*. *muris* interferes with hMDMs’ Mtb Ag-presentation to Mtb Ag-specific CD4^+^ T cells

To elucidate whether the immunomodulatory effects of the individual helminth antigens affected hMDMs ability to present Mtb-antigens we generated Mtb Ag-specific CD4+ T-cells which were cultured with autologous hMDMs. As helminth antigens were seen to affect the proliferation of Mtb inside macrophages (Fig 6), and the Mtb Ag-specific presentation would be influenced by the number of bacteria for the net availability of mycobacterial antigens, these experiments were performed with inactivated *M*. *tuberculosis* H37Rv thereby keeping the source of antigen (e.g. the bacteria) equal throughout the treatments. *H*. *diminuta* and *T*. *muris* exposed and infected hMDMs co-cultured with autologous PPD- or Ag85B-specific CD4^+^ T cells significantly reduced the Mtb-induced IFN-γ secretion by the Mtb Ag-specific CD4^+^ T cells (Fig 7A and 7B). *S*. *mansoni* antigen exposure of hMDMs did not affect their capacity to stimulate the Mtb Ag-specific CD4^+^ T cells. The positive control SEB markedly induced IFN-γ, whereas the negative control ovalbumin did not induce any IFN-γ production above that of uninfected hMDMs. Besides the helminth-driven skewing effect in antigen presentation (i.e. IFN-γ release from Mtb Ag-specific CD4^+^ T cells) when hMDMs were stimulated with intact Mtb bacteria (Fig 7), *H*. *diminuta* pre-exposed hMDMs were further seen to also reduce the release of the Th1-cytokines TNF-α and IL-2 upon mycobacterial protein stimulation (S1 Fig). In agreement with intact bacterial stimulated hMDMs, both *H*. *diminuta* and *T*. *muris* exposed hMDMs reduced the IFN-γ release from Mtb Ag-specific CD4^+^ T cells when mycobacterial proteins were used for stimulating the hMDMs.

### Autophagosome maturation is reduced in *H*. *diminuta* and *T*. *muris* co-exposed hMDMs

Acidification of the phagosome contributes to degradation of bacteria and generation of bacterial peptides delivered for antigen presentation [37]. Since autophagy is involved in delivering antigens to the MHC class-II loading compartment [37], we tested if the helminth antigens affected autophagy in Mtb infected hMDMs (Fig 8). With a 48h helminth antigen pretreatment alone the antigens did not significantly affect the autophagy proteins LC3B and SQSTM1. However, *H*. *diminuta* and *T*. *muris*-antigen pretreatment and Mtb co-exposure markedly enhanced accumulation of LC3BII, and significantly accumulated the autophagy substrate SQSTM1 (p < 0.05 for both *H*. *diminuta* and *T*. *muris*-antigen treatment), compared to unexposed Mtb infected hMDMs. Buildup or accumulation of autophagy proteins in hMDMs during infection with *M*. *tuberculosis* is caused by *M*. *tuberculosis* blocking autophagosome maturation of the bacteria containing vacuoles as previously shown [28]. The presented data indicate that co-exposure with helminth derived antigens further obstruct a functional autophagy, needed for both elimination of the bacteria and generation of Mtb-antigens for MHC class-II loading.

## Discussion

There are conflicting data regarding the interplay between helminth and Mtb infections, with some studies showing an increased bacterial burden in co-infected animals [7,8,19], while others show no effect [38–40] or a decreased burden of mycobacteria [9,41]. We hypothesized that helminths would make the hMDMs less capable of controlling Mtb infection, and used antigens from three distinct groups of helminths to investigate this. It is important to note that the effects documented here are in the absence of T cells (or any other cell of the adaptive immune response). We found that antigens from different helminths cause different responses against Mtb in human macrophages with respect to lysosome function, macrophage polarization, Mtb burden and antigen processing of Mtb. Antigens from the nematode *T*. *muris* and the cestode *H*. *diminuta* induced similar responses in hMDMs leading to an increased burden of Mtb, while soluble egg antigens from the trematode *S*. *mansoni* induced a response that favored the host by decreasing Mtb burden. This was consistent with the decreased phagosome maturation seen in hMDMs co-exposed to *H*. *diminuta* and *T*. *muris* antigen and the unaffected Mtb-phagosome acidification with *S*. *mansoni* soluble egg antigen.

When exposing the macrophages to antigen directly prior to Mtb infection, the macrophage burden of Mtb was largely unchanged independent of the helminth antigen used. However, upon 48h pretreatment (mimicking a chronic infection) with the helminth antigens, an increase in the Mtb burden could be seen upon *H*. *diminuta* and *T*. *muris* antigen treatment while exposure to soluble egg antigens from *S*. *mansoni* increased the control of Mtb. The production of cytokines was accordingly more pro-inflammatory at early exposure time-points and shifted to a more anti-inflammatory response, with increased IL-10, with a longer exposure to *T*. *muris* and *H*. *diminuta* antigens, while *S*. *mansoni* antigens decreased the IL-10 production from Mtb-infected hMDMs. This indicates that some helminths prime the innate immune response towards a more pro-inflammatory response while others push it towards an anti-inflammatory response which could affect the outcome of a bacterial infection that usually is dominated by a Th1 pro-inflammatory response. This is in accordance with another study showing that *T*. *muris* evoked an increase in pro-inflammatory cytokines such as TNF-α along with an increase of IL-4 and IL-10 compared to BCG infection and co-infection [38]. This initial pro-inflammatory response may be due to the activation of TLR4 by the glycans from the helminths, as shown by Goodridge *et al* [42]. However it seems that after a longer infection period with helminths, the response becomes dominated by Th2 cytokines with a concurrent decrease in Th1 cytokines [43].

As seen previously, helminths can induce AAMs with increased expression of arginase-1 in mice, which can be less capable of combating infection with bacteria [10,19,43,44], although this kind of type 2 response is needed to expel the helminths [21]. IL-10 could promote the development of these alternative activated macrophages [44]. This cytokine is also associated with a higher sensitivity to Mtb in mice, causing increased bacterial load and mortality [44]. Similarly, we observed polarization towards a M2-type macrophage in *H*. *diminuta* and *T*. *muris* antigen-treated hMDMs, with elevated CCL22 expression (specifically expressed during M2-polarization; [36]), increased IL-10 levels, and reduced capacity to combat Mtb. On the other hand, *S*. *mansoni* treated hMDMs expressed higher levels of CCR7 (M1 macrophages; [35]), produced less IL-10 during infection, showed no elevation in CCL22 and were also more fit to control Mtb. A study with *T*. *muris* and Mtb co-infected mice showed no effect on the bacterial load, although there was a decreased response to both pathogens which lead to delayed expulsion of the helminth and a decreased Th1 response in the lung [38].

A previous study showed reduced expression of co-stimulatory molecules in dendritic cells and macrophages along with decreased numbers of IL-10 and IFN-γ producing CD4^+^ T cells upon *Brugia malayi* microfilariae exposure [45]. During moderate Mtb co-exposure (MOI 1–5), we observed that the *T*. *muris* antigen induced higher IL-10 levels than *H*. *diminuta*, and that *H*. *diminuta* antigen stimulation of hMDMs enhanced their CCL22 expression in the absence of Mtb but that *T*. *muris* antigen required co-exposure with Mtb for CCL22 expression. To further investigate whether the helminth antigens polarized hMDMs into different AAM phenotypes additional markers were employed. One of the markers investigated here was CD206 (the mannose receptor) and expression of this marker indicates a M2a phenotype in humans and mice [3,35]. Besides the increase in IL-10, we observed that *T*. *muris* treated macrophages had increased CD206 expression indicative of a M2a-like macrophage. The mannose receptor has been found to be important in the binding of *T*. *muris* antigen and uptake of *S*. *mansoni* antigens by macrophages, causing a reduced production of pro-inflammatory cytokines [20,21]. In our study only *T*. *muris* treated hMDMs had increased expression of CD206 which was not seen in *S*. *mansoni* co-exposed hMDMs. The expression of DC-SIGN was also increased in hMDMs co-exposed to *T*. *muris* indicative of a regulatory M2-like macrophage. This receptor is induced on alveolar macrophages during Mtb infection and its expression has been associated with increased susceptibility to Mtb, since the pathogen binds more easily to the macrophages [46]. The Mtb burden was highest in the *T*. *muris* co-exposed hMDMs, possibly due to the high IL-10 production and the lack of IL-1β compared to *H*. *diminuta* co-exposed cells that exhibited an augmented IL-1β production and a less dramatic effect on Mtb replication. Furthermore, during co-infection with Mtb these antigens promoted different types of regulatory macrophages: *T*. *muris* antigen increased CD206 expression (M2a-like macrophages) whereas *H*. *diminuta* antigen increased CD163 expression (M2c-like macrophages). Infection with helminths (as modeled here by helminth antigens) will impact how the host deals with TB, and as we show this is a complex and species-specific interaction such that in the field it will be important to determine not only if the individual is Th2 skewed but also if the helminth-infection is a trematode, a cestode or a nematode as well as determining the species, since it is likely that there will be a species-specific differential effect on the outcome of TB.

Several studies have shown an impact on the polarization of the adaptive immune response upon helminth and mycobacterial co-infection, with reduced levels of Th1 cytokine expressing T cells [17,18,38,45] and increased levels of regulatory T cells [47,48]. To further elucidate the response towards Mtb during co-exposure to helminth antigens in hMDMs, Mtb-antigen presentation was measured by the activation of Mtb antigen-specific CD4^+^ T cells. hMDMs co-exposed to Mtb and antigen from *H*. *diminuta* or *T*. *muris* caused less activation of the CD4^+^ T cells, indicating reduced efficiency in Mtb-antigen presentation by the hMDMs to the T cells. Together with the reduced LysoTracker co-localization to Mtb and the accumulation of autophagy proteins, this implies deficient processing of Mtb antigens in the co-exposed hMDMs that would lead to a decreased activation of CD4^+^ T cells. In contrast, hMDMs co-exposed to Mtb and antigen from *S*. *mansoni* did not lead to reduced T cell activation or reduced LysoTracker co-localization, which is in accordance with the increased control of Mtb. However, this is in contrast to another study showing that *S*. *mansoni* antigen impaired Mtb specific T cell responses with a reduction of IFN-γ and reduced control of Mtb [19]. The reason for the contradictory results might be due to the fact that the first response to a schistosoma infection is dominated by Th1 events, while the production of eggs later during infection causes a shift towards a Th2 response [49]. Additionally, the differences between other studies and data herein are that we assessed the direct effect of helminth antigens on macrophages without the involvement of a Th2 response. A recent example of helminth exposure of Mtb-specific T cells, showed that *S*. *mansoni* soluble antigen exposed T cells of TB infected individuals produced increased levels of anti-inflammatory IL-10 that caused a phagosomal arrest in Mtb infected human macrophages [50].

In conclusion, our study shows that different helminth antigens can have direct effects on macrophages and cause different responses to Mtb in co-exposed hMDMs. *H*. *diminuta* antigens and to a greater degree *T*. *muris* antigens caused an anti-inflammatory response with M2-type polarization and increased IL-10 secretion, along with decreased T cell activation, in Mtb infected cells. These co-exposed hMDMs also exhibited reduced bactericidal functions as shown by reduced phagosome maturation and an increased Mtb burden. Antigen from *S*. *mansoni* had the opposite effect on macrophages, causing a decrease in IL-10 output, a M1-type polarization and an increased control of Mtb. As expected the interaction of helminths (mimicked by use of helminth antigens) and Mtb is complex and species-specific and while the mechanism(s) of this trans-kingdom interaction need to be fully defined, it is clear that in helminth-endemic areas the outcome of TB will be influenced by the helminth burden. Assuming the *in vitro* data presented herein translate to infected humans the challenge will be to develop effective therapy for TB that considers the patients co-infection status.

## Supporting information

S1 Fig Mycobacterial protein-stimulated hMDMs pre-exposed to *H*. *diminuta* decreases Th1-cytokine secretion from Mtb-antigen specific CD4+ T cells. hMDMs were left untreated, or treated for 48h with 10 μg/ml of *H*. *diminuta* (H.d), *T*. *muris* (T.m), or *S*. *mansoni* soluble egg antigen (S.m). Thereafter hMDMs were stimulated with purified protein derivative (PPD; culture filtrates from Mtb strain H37Rv) for 24h, before being co-cultured with autologous PPD-specific (left) or Ag85B-specific (right) CD4^+^ T cells (1:5 DC:T cell ratio). Cell free culture supernatants were collected 48h later, and assayed for IFN-γ, TNF-α, and IL-2, data expressed as means ± SEM from 7 independent hMDM donors. p*<0.05, p**<0.01 using One-way ANOVA.
(TIF)
