# Supplementary figures and images for: Species dependent impact of helminth-derived antigens on human macrophages infected with Mycobacterium tuberculosis: Direct effect on the innate anti-mycobacterial response
Source: PLoS Negl Trop Dis. 2017 Feb 13;11(2):e0005390. doi: 10.1371/journal.pntd.0005390 (PMC5325601; doi:10.1371/journal.pntd.0005390)

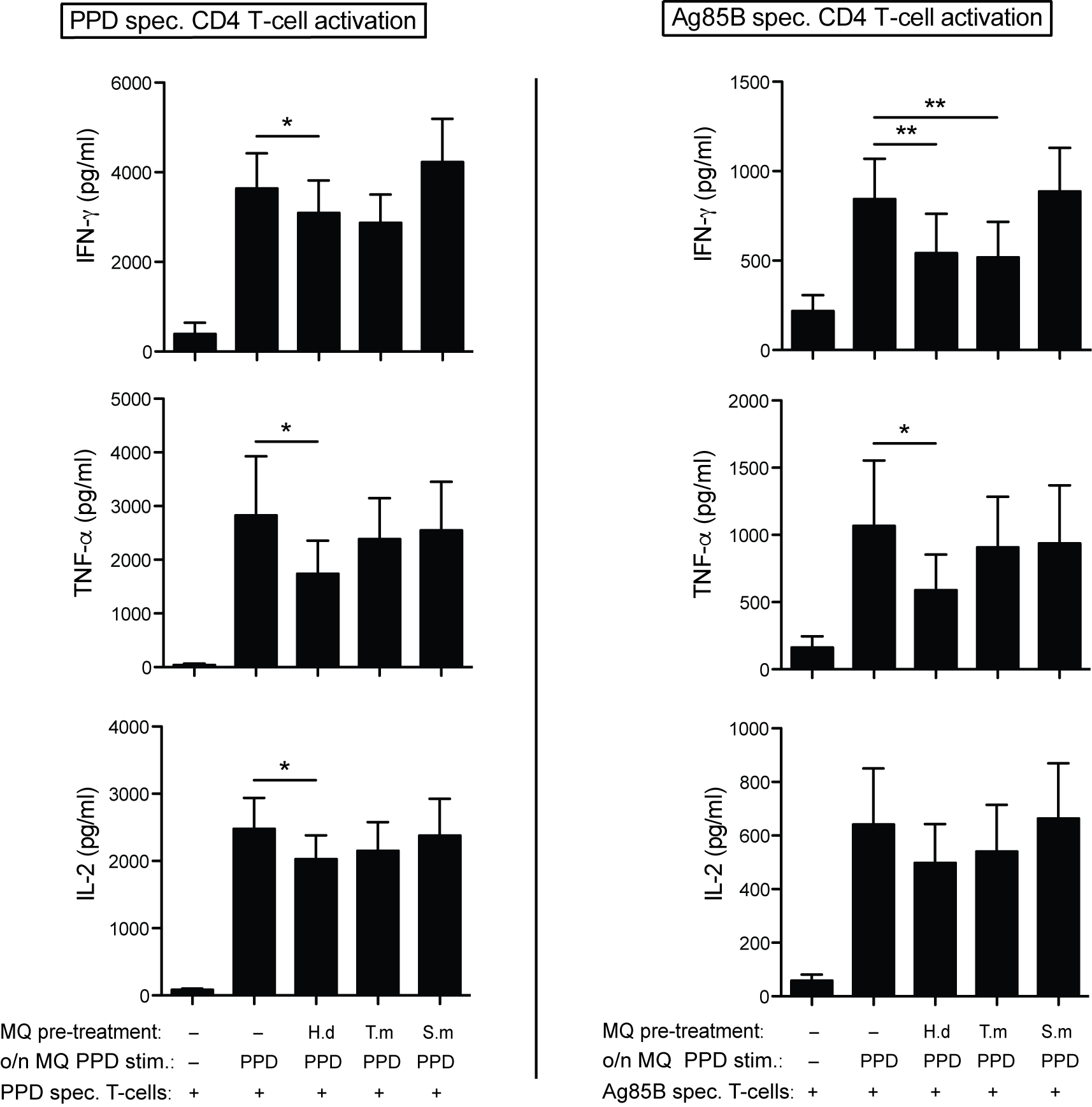

Supplement: S1 Fig — hMDMs were left untreated, or treated for 48h with 10 μg/ml of H. diminuta (H.d), T. muris (T.m), or S. mansoni soluble egg antigen (S.m). Thereafter hMDMs were stimulated with purified protein derivative (PPD; culture filtrates from Mtb strain H37Rv) for 24h, before being co-cultured with autologous PPD-specific (left) or Ag85B-specific (right) CD4+ T cells (1:5 DC:T cell ratio). Cell free culture supernatants were collected 48h later, and assayed for IFN-γ, TNF-α, and IL-2, data expressed as means ± SEM from 7 independent hMDM donors. p*<0.05, p**<0.01 using One-way ANOVA. (TIF) [file pntd.0005390.s001.tif]
